# Yoga and Hypertension

**DOI:** 10.4172/2157-7595.1000144

**Published:** 2013-11-13

**Authors:** Debbie L Cohen

**Affiliations:** Perelman School of Medicine at the University of Pennsylvania, Renal, Electrolyte and Hypertension Division, USA

Hypertension is a major public health issue affecting more than 70 million US adults and is a major risk factor in the development of stroke, cardiovascular [CV] and chronic kidney disease [1]. Patients with prehypertension [BP 130-130/80-89 mmHg] [2] are also at an increased risk for adverse CV events compared to normotensive controls [3]. Lifestyle modifications [LSM] have been recommended as a first line approach for both prehypertension and stage 1 hypertension patients [2]. Complementary and Alternative Medicine [CAM] modalities including mind-body therapies have been used in managing modest elevations in BP [4]. Yoga has been shown to be one of the most popular CAM therapies with growing use particularly in older hypertensive patients [5-8]. Although yoga has been beneficial in treating a variety of medical conditions [9,10] there has been limited data published suggesting a benefit of yoga on hypertension.

Although there are a number of published studies investigating the effects of various forms of yoga on hypertension [4,11-23], most of these studies are uncontrolled case reports or small cohort studies conducted in India with significant methodological limitations. There are only 9 randomized controlled trials [RCT] of any form of yoga for hypertension [11,13,15-21,23]. They are shown in Table 1. This includes our own previous study completed at the University of Pennsylvania in which subjects with prehypertension to stage 1 hypertension were randomized to a structured Iyengar Yoga [IY] Program or enhanced LSM. This study showed clinically meaningful reductions in 24-hour ambulatory BP readings in the IY group at 12 weeks [17]. The most recent publication from Canada randomized unmedicated stage 1 hypertensive patients to meditation and yoga for 8 weeks versus wait-list control [20]. Ambulatory BP monitoring was performed at baseline and at 12 weeks. Results of ABP did not lower BP by a statistically or clinically meaningful amount in the treatment group as opposed to the control group. Another recent study from St Louis of 60 HIV infected adults with moderate CVD and with 83% of subjects with hypertension were assigned to 20 weeks of supervised yoga practice or standard of care treatment. Resting SBP and DBP improved more in the yoga group than in the standard of care group [21]. A recent study from India was a cross-over RCT of an earlier RCT of non-pharmacological interventions in hypertension. Subjects were prehypertensive adults who were randomly allotted to a group that they had not been in the previous trial [19]. They were assigned to one of 4 groups for 8 weeks: control group, brisk walking 50-60 minutes 3-4 days a week, sodium restriction to at least half of their previous intake or yoga for 30-45 minutes per day, 5 days per week. All 3 groups aside from the control group showed reduction in BP but physical exercise showed a greater reduction on BP in the range of 5/6 mm Hg whereas sodium reduction and yoga showed a less impressive decline in BP in the range of 2.5/2.0 mm Hg [18]. Another study involved 101 subjects with features of the metabolic syndrome who were randomized to standard medical therapy or yoga and a form of transcendental meditation. SBP was reduced significantly by 16 mm Hg in the treatment group [23]. Another prior study was an 8 week pranayama and asana yoga program conducted in 27 untreated hypertensive patients and 27 controls living in Tailand [11]. The experimental group significantly reduced SBP by 25 mm Hg at 8 weeks compared to 2 mm Hg increase in the control group. DBP significantly decreased by 18 mm Hg in the experimental group compared to an increase of 2 mm Hg in the control group. In India, 33 hypertensive adults were randomly assigned to 3 groups [yoga, medications only, or no therapy] and were followed for 11 weeks [15]. Yoga was performed at home for 6 hours per week and included a combination of asanas, pranayamas and mantras. At the end of the study, SBP was reduced by an impressive 33 mmHg compared to 4 mmHg in the control group and 24 mmHg in the poorly described drug therapy group. The differences were significant compared to both control and drug treatment. In an older randomized controlled trial from England, 43 patients with known hypertension, most of whom were already medically treated, were randomized to yoga plus biofeedback or usual care [16]. Treatment reduced SBP by 26 mmHg vs. 9 mmHg in the control. This study however used a mixed intervention which included biofeedback in addition to yoga, and did not include any movement.

A general critique of the published yoga and hypertension research includes the fact that most studies were not randomized, had inadequately described yoga or control programs, did not collect information on other lifestyle confounders [e.g. adoption of vegetarian diet, reduction in alcohol intake] and did not use standardized, reliable outcomes measures. There is also the very real possibility of publication bias in which negative yoga trials have not been published. Some of the older studies also report much more impressive reductions in BP than one would expect with a lifestyle intervention and this does question the validity of the data. Most lifestyle interventions including regular aerobic exercise, dietary sodium reduction and weight loss of 10 kg usually result in a BP reduction in the range of 4-10 mm Hg in SBP.

The effects of yoga on lowering blood pressure in more recent studies have mostly been modest however data from the Framingham Heart Study showed that a 2 mm Hg reduction in DBP could reduce the risk of stroke or transient ischemic attack by 14% [24]. While a 10 mm Hg reduction in SBP, seen with prescription drugs and in some meditation studies [25], is associated with a 30% relative reduction in risk of stroke [26]. Thus smaller reductions in BP [5 mm Hg in SBP or 2 mmHg in DBP] achievable through diet, some dietary supplements and mind body therapies can be expected to significantly reduce CVD morbidity.

A recent meta-analysis was conducted of 17 randomized and non-randomized trials of yoga and hypertension. Results showed that yoga had a modest effect on both SBP [-4.17 mm Hg] and DBP [- 3.26 mm Hg]. There was substantial heterogeneity present across the included studies. The effects of yoga on BP varied by the type of yoga intervention and by comparison group but not by duration of yoga practice. When the analysis was restricted to studies using interventions incorporating three elements of yoga practice[postures, meditation and breathing] larger reductions of SBP and DBP [-8.17 mmHg and-6.14 mmHg] were observed. Yoga was also associated with a significant decline in SBP and DBP [-7.96 mm Hg and -5.52 mm Hg] relative to no treatment but not when compared to exercise or other intervention types [27].

We are currently completing a randomized clinical trial of yoga utilizing gold standard methodologies in the measurement of BP to rigorously evaluate the efficacy of yoga in subjects with prehypertension and stage 1 hypertension. Enrollment goal is 120 subjects. Subjects are randomized to 1 of 3 treatment groups: yoga practice in a studio 2-3 × per week for 24 weeks versussupervised diet/weight reduction and walking program versus combination program consisting of both yoga and the dietary/walking program intervention. Subjects have inpatient ambulatory BP monitoring at baseline, 12 and 24 weeks [28]. The study will be completed by December 2013.

There is a genuine need for rigorously conducted randomized clinical trials of yoga assessing the effects of lowering BP in patients with prehypertension and stage 1 hypertension. Even if the effects of yoga on hypertension are modest this can still provide substantial CV protection for this group of patients with mild to moderate hypertension and may afford patients the opportunity to engage in yoga instead of committing to lifelong antihypertensive medication.

## Figures and Tables

**Table 1:** Randomized controlled trials of yoga and hypertension.

Study Author	Country Journal/year	Subject #	Intervention	BP Outcomes [mm Hg]	Summary
Patel [16]	England Lancet 1975	43 subjects with hypertension	Yoga vs. usual care	SBP decrease: Yoga: -26 Usual care: -9	Large reduction in SBP
Murugesan et al. [15]	India Indian Journal of Physiology 2000	43 subjects with hypertension	3 groups 1. Yoga 2. Medication 3. No treatment Yoga home practice 6 hrs per week × 11 weeks	SBP decrease: 1: -33 2: -24 3: -4	Large reduction in SBP
McCaffrey et al. [11]	Thailand Holistic Nursing Practice 2005	27 untreated hypertensive patients and 27 controls	Yoga for 8 weeks versus control	SBP/DBP change Yoga: -25/-18 control: +2/+2	Large reduction in SBP
Khatri et al. [23]	India India Diabetes Clinical Practice 2007	101 subjects	Medical treatment versus yoga and meditation	Reduction of SBP by 16 in yoga group	Large reduction in SBP
Saptharishi et al. [19]	India Indian Journal of Medicine 2009	113 subjects with prehypertension and hypertension	102 completed 1 – control 2- exercise 3 – low sodium 4 – yoga	1 – no change 2 – 5.3/6.0 3 – 2.6/3.7 4 – 2.0/2.6	Exercise more effective than salt or yoga
Subramanian et al. [18]	India Indian Journal of Medicine 2011	98 subjects	94 completed 1 – control 2- exercise 3 – low sodium 4 – yoga	SBP/DBP change 1: no change 2: - -5.3/-6.0 3: -2.5/-2.0 4: -2.3/-2.4	Exercise more effective than sodium reduction or yoga
Cade et al. [21]	St Louis, USA HIV Medicine 2010	60 HIV subjects with mild/moderate CV disease risk	20 weeks of supervised yoga or standard care	SBP/DBP change Yoga: -5/-3 Standard care: +1/+2	Yoga shows modest reduction in SBP and DBP
Blom et al. [20]	Canada	Prehypertension and stage 1 hypertension	Meditation and yoga vs. wait list control × 8 weeks	Mean awake ABP and 24 hr ABP	No significant change in BP in either group
Cohen et al. [17]	Philadelphia, USA Evidence Based Complementary and Alternative Medicine 2011	78 patients with prehypertension and stage 1 hypertension 32 – Diet group 46 – Yoga group	31 subjects completed diet intervention and 26 completed the yoga intervention. Diet versus yoga for 12 weeks	24 hr ABP: SBP/DBP change Diet: -4/-2 Yoga: -6/-5	Modest reduction in SBP and DBP
Cohen et al. [28]	Contemporary Clinical Trials 2013	120 subjects with prehypertension and stage 1 hypertension	1. Diet/walking 2. Yoga 3. Yoga and diet Study conducted over 24 weeks	24 hr ABP	Will be completed in 12/13.

ABP = ambulatory BP, SBP = systolic BP, DBP = diastolic BP
